# Observation of a new type of self-generated current in magnetized plasmas

**DOI:** 10.1038/s41467-022-34092-0

**Published:** 2022-10-29

**Authors:** Yong-Su Na, Jaemin Seo, Yoonji Lee, Gyungjin Choi, Minseo Park, Sangjin Park, Sumin Yi, Weixing Wang, Min-Gu Yoo, Minsoo Cha, Beomsu Kim, Young-Ho Lee, Hyunsun Han, Boseong Kim, Chanyoung Lee, SangKyeun Kim, SeongMoo Yang, Cheol-Sik Byun, Hyun-Seok Kim, Jinseok Ko, Woochang Lee, Taik Soo Hahm

**Affiliations:** 1 Department of Nuclear Engineering, Seoul National University, Seoul, 08826 Republic of Korea; 2 Princeton University, Princeton, NJ 08544 USA; 3 Korea Institute of Fusion Energy, Daejeon, 305-333 Republic of Korea; 4 Princeton Plasma Physics Laboratory, Princeton, NJ 08540 USA; 5 General Atomics, San Diego, CA 85608 USA

**Keywords:** Magnetically confined plasmas, Nuclear fusion and fission

## Abstract

A tokamak, a torus-shaped nuclear fusion device, needs an electric current in the plasma to produce magnetic field in the poloidal direction for confining fusion plasmas. Plasma current is conventionally generated by electromagnetic induction. However, for a steady-state fusion reactor, minimizing the inductive current is essential to extend the tokamak operating duration. Several non-inductive current drive schemes have been developed for steady-state operations such as radio-frequency waves and neutral beams. However, commercial reactors require minimal use of these external sources to maximize the fusion gain, Q, the ratio of the fusion power to the external power. Apart from these external current drives, a self-generated current, so-called bootstrap current, was predicted theoretically and demonstrated experimentally. Here, we reveal another self-generated current that can exist in a tokamak and this has not yet been discussed by present theories. We report conclusive experimental evidence of this self-generated current observed in the KSTAR tokamak.

## Abstract (web summary)

Fusion devices like tokamaks require plasma current to generate magnetic field for plasma confinement. Here the authors report an observation of a self-generated anomalous current that contributes up to 30% of the total current in the fusion plasma at KSTAR.

## Introduction

Nuclear fusion has drawn more attention in the era of carbon neutrality because of no carbon dioxide production during power generation and no generation of high-level radioactive wastes. For the economic competitiveness of a nuclear fusion reactor, steady-state operations with high availability are required. For decades since the invention of the tokamak, various external or self-generated current drive mechanisms have been developed for steady-state operations, such as radio frequency-driven, neutral beam injection (NBI)-driven, and bootstrap current. Recently, it was found that micro-instabilities can also generate or modify the plasma current^1–4^, which could probably explain an anomaly in the current profile found in experiments such as in the hybrid operation scenario^5^. The self-generated current drive mechanisms have been predicted in theories^1,2,4,6^, then discovered in experiments^7–9^ and they were found to be consistent with each other.

In this work, we present an experimental finding of a new type of self-generated current drive source in the KSTAR tokamak, which is not explained by the theories of existing current drive mechanisms. The observed self-generated current accounts for ~30% of the total plasma current, appearing in the off-axis region of the plasma.

## Results

### Evidence of anomalous current source in KSTAR

**Fig. 1:**
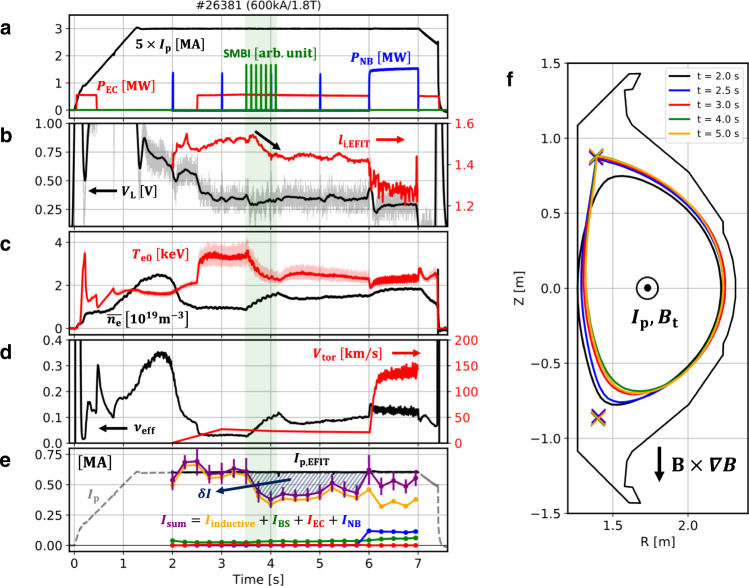
Overview of main parameters of shot #26381 in KSTAR. **a** Plasma current (${{I}}_{{{{{{\rm{p}}}}}}}$), neutral beam injection (${{P}}_{{{{{{\rm{NB}}}}}}}$) and electron cyclotron (${{P}}_{{{{{{\rm{EC}}}}}}}$) heating power, and supersonic molecular beam injection (SMBI). **b** Loop voltage (${{V}}_{{{{{{\rm{L}}}}}}}$) and internal inductance (${{l}}_{{{{{{\rm{i}}}}}}}$) from EFIT. **c** Central electron temperature (${{T}}_{{{{{{\rm{e}}}}}}{{{{{\rm{0}}}}}}}$) and line-averaged density ($\bar{{{n}}_{{{{{{\rm{e}}}}}}}}$). **d** Effective collisionality (${{\nu }}_{{{{{{\rm{eff}}}}}}}$) and central toroidal rotation velocity of the carbon impurity (${{V}}_{{{{{{\rm{tor}}}}}}}$). **e** Components of the plasma current by interpretive simulations; summation of each component of current (${{I}}_{{{{{{\rm{sum}}}}}}}$), inductive (${{I}}_{{{{{{\rm{inductive}}}}}}}$), bootstrap (${{I}}_{{{{{{\rm{BS}}}}}}}$), electron cyclotron (${{I}}_{{{{{{\rm{EC}}}}}}}$), and neutral beam (${{I}}_{{{{{{\rm{NB}}}}}}}$) current. **f** Plasma boundary configuration and X-points in #26381. The *X*-point is the unfavorable direction with respect to ${B}{\times }{\nabla }{B}$. The plasma current and the toroidal magnetic field are in the direction out of the paper. The time period with SMBI is highlighted with a light green shade.

An example of dedicated experiments in KSTAR^10^, a Deuterium discharge, shot #26381, is introduced in Fig. 1. This experiment was designed with the purpose of minimizing both the external current drive and the known self-generated current drive in order to make it easy to identify a possible new current source; (i) the external current drive is minimized where electron cyclotron (EC) wave, the only external source, was injected in the nearly perpendicular direction to heat up the plasma but to avoid the current drive in the toroidal direction and (ii) the L-mode confinement regime was maintained by avoiding the H-mode transition with the aid of an unfavorable magnetic field null configuration as shown in Fig. 1f so as to reduce the bootstrap current.

The main parameters presented in Fig. 1 are as follows; the toroidal magnetic field ${B}_{{\rm {t}}}=1.8\,{{{{{\rm{T}}}}}}$, the plasma current ${I}_{{\rm {p}}}=0.6\,{{{{{\rm{MA}}}}}}$, the major radius $R=1.78\,{{{{{\rm{m}}}}}}$, and the minor radius $a=0.46\,{{{{{\rm{m}}}}}}$. The heating power by EC heating and NBI were ${P}_{{\rm {{EC}}}}=0.6\,{{{{{\rm{MW}}}}}}$ and ${P}_{{\rm {{NB}}}}=1.5\,{{{{{\rm{MW}}}}}}$, respectively. The electron density (${n}_{{{{{{\rm{e}}}}}}}$) and temperature (${T}_{{{{{{\rm{e}}}}}}}$) were measured by Thomson scattering^11^ and calibrated by the interferometry^12^ and electron cyclotron emission (ECE)^13^ diagnostics, respectively. The ion temperature (${T}_{{{{{{\rm{i}}}}}}}$) was measured by charge exchange spectroscopy (CES)^14^. Note that the total plasma current (${I}_{{{{{{\rm{p}}}}}}}$) was feedback controlled during the whole discharge by adjusting the toroidal loop voltage (${V}_{{{{{{\rm{L}}}}}}}$) induced by the primary coil.

In shot #26381, the plasma boundary was diverted at $t=2.0\,{{{{{\rm{s}}}}}}$ and almost invariant after $t=3.0\,{{{{{\rm{s}}}}}}$. At $t=3.5\,{{{{{\rm{s}}}}}}$, supersonic molecular beam injection (SMBI)^15^ was applied so that the electron density was increased and the electron temperature decreased. For comparison, EC was replaced by NBI in $6.0\,{{{{{\rm{s}}}}}}\le t\le 7.0\,{{{{{\rm{s}}}}}}$. After NBI was applied, sawtooth instability appeared, whereas no magneto-hydrodynamic instability was noticeable before $t=6.0\,{{{{{\rm{s}}}}}}$.

As the electron density was increased by SMBI at $t=3.5\,{{{{{\rm{s}}}}}}$ in #26381, the core electron temperature was decreased, as shown in Fig. 1c. This resulted in an increase in plasma resistivity ($\eta \propto {T}_{{{{{{\rm{e}}}}}}}^{-1.5}$). Since the loop voltage induced by the primary coil was almost constant in this phase, the inductive current should have been reduced. Figure 1e shows each component consisting of the plasma current obtained by the interpretive simulation with an integrated tokamak simulator, TRIASSIC^16^. Before $t=3.5\,{{{{{\rm{s}}}}}}$, the summation of each component (purple line) agrees with the total plasma current measured by the Rogowski coil (gray dashed line) as well as the EFIT^17^ reconstruction with a constraint of motional Stark effect (MSE) measurement^18^ (black solid line), within the uncertainty range estimated from the standard deviation of the loop voltage signals. However, as the inductive current becomes reduced due to the increase of the resistivity by SMBI at $t=3.5\,{{{{{\rm{s}}}}}}$, an anomalous current gap ($\delta I$ in Fig. 1e) begins to appear between the simulation and the experiment. This is in contrast to the behavior at $t=2.5\,{{{{{\rm{s}}}}}}$, where the interpretive simulation matches the experiment even though the electron temperature significantly changes. Here, it is noteworthy that the EC-driven and the bootstrap current are below 5% of the total plasma current due to the perpendicular EC injection and low poloidal beta (${\beta }_{{{{{{\rm{p}}}}}}} \, < \,0.3$) with the L-mode confinement, respectively. This discrepancy between the simulation and the experiment is sustained for longer than 2 s until NBI was applied which is longer than the current diffusion time, ${\tau }_{{{{{{\rm{r}}}}}}}=\frac{{\mu }_{0}{a}^{2}}{2\eta } \sim$ 0.5 s in this experiment. It indicates a possibility of an additional steady source of the plasma current, rather than a transient phenomenon by unsaturated current diffusion. The internal inductance (${l}_{{{{{{\rm{i}}}}}}}$), an indicator of the current density profile peaking, was reduced as this anomalous current appears (see Fig. 1b) even though external off-axis current drives were negligible in those discharges, implying that this anomalous current might appear in the off-axis region, clearly distinguished from the inductive current which is concentrated in the on-axis region of the plasma. Note that ${l}_{{\rm {i}}}$ can also be reduced while the inductive current is decreased. The location where the anomalous current strongly appears will be discussed later.

To investigate the contribution of ions to this current, we checked the time evolution of the ion rotation. A previous study^19^ showed that the intrinsic rotation of ions could be generated in plasmas and its characteristics are strongly affected by the effective collisionality. The intrinsic rotation of the ions can induce the toroidal current even though the ion contribution is usually smaller than the electron. Figure 1d shows the toroidal rotation velocity of the Carbon ion at the center of the plasma (${V}_{{{\rm {tor}}}}$) measured by CES and the corresponding effective collisionality, ${\nu }_{{{{{{\rm{eff}}}}}}}\equiv 0.1{Z}_{{{{{{\rm{eff}}}}}}}R{n}_{{{{{{\rm{e}}}}}}}/{T}_{{{{{{\rm{e}}}}}}}^{2}$, where ${Z}_{{{{{{\rm{eff}}}}}}}$ is the effective charge and $R$ is the major radius. However, the ion rotation observed during the anomalous current phase ($t=3.5-6.0\,{{{{{\rm{s}}}}}}$) in #26381 does not change significantly due to SMBI. Therefore, the anomalous current is thought to be contributed by electrons dominantly.

**Fig. 2:**
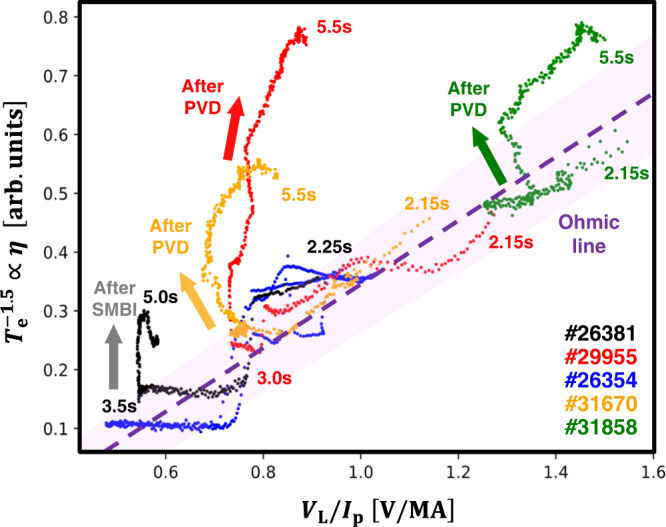
Time trajectory of ${{T}}_{{\rm {e}}}^{-1.5}$ versus *V*_L_/*I*_P_ in #26381, #29955, #26354, #31670, and #31858. The purple-shaded line represents the proportional line.

This phenomenon was also observed in other experiments in KSTAR where the fueling was replaced from SMBI to the gas fueling at the diverter region by the piezoelectric valve at D-port (PVD); shot #29955, #31670, and #31858. The discharge, #31858 was conducted without EC, a pure ohmic discharge. In Fig. 2, it is apparent that the relation between ${T}_{{{{{{\rm{e}}}}}}}^{-1.5}\,\left(\propto \eta \right)$ and ${V}_{{{{{{\rm{L}}}}}}}/{I}_{{{{{{\rm{p}}}}}}}$ in #26381, #29955, #31670, and #31858 diverges from the typical ohmic relation after the fueling with SMBI or PVD. It is noteworthy that a similar discharge but without any fueling, #26354, does not show such divergence. The amount of the anomalous current is found to be ~30% of the total current, which is comparable to the current driven by one NBI source between 6.0 and 7.0 s shown in Fig. 1e. After NBI was applied instead of EC at $t=6.0\,{{{{{\rm{s}}}}}}$, however, the anomalous current almost disappeared.

**Fig. 3:**
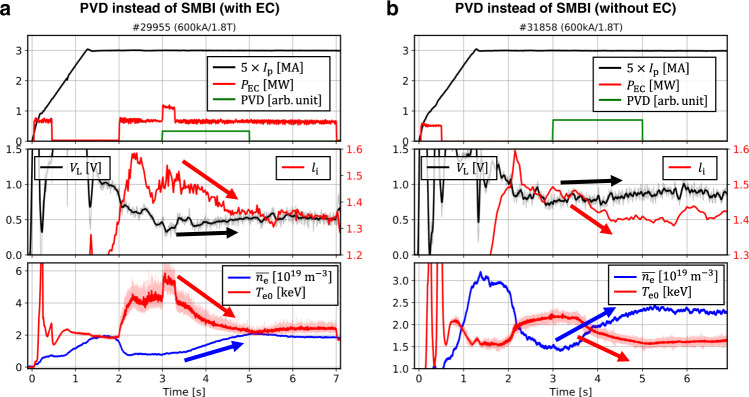
Overview of main parameters. #29955 (**a**) and #31858 (**b**), where anomalous currents were also observed. Here, gas fueling at the diverter region by the piezoelectric valve at D-port (PVD) was used instead of supersonic molecular beam injection (SMBI). The discharge, #31858 is a pure Ohmic discharge without EC.

Figure 3 shows two different discharges, #29955 (a) and #31858 (b), where anomalous current drives were also observed. In both discharges, PVD instead of SMBI increased the plasma density from $t=3.0$ to $5.0\,{{{{{\rm{s}}}}}}$. However, the induced loop voltage remained almost constant even though the electron temperature and the plasma resistivity significantly changed. ${l}_{\rm {{i}}}$ was also reduced in the same manner as #26381 shown in Fig. 1b and remained for 2.0 s after the fueling was turned off. This indicates that the observation of the anomalous current drive is not due to a special effect caused by SMBI alone and seems to be related to the level of the density or the collisionality. Furthermore, this phenomenon was consistently observed in #31858, the pure ohmic discharge shown in Fig. 3b. This can exclude the possibility of an error from mis-estimating the EC-driven current drive depending on the collisionality regime^20^ or any unknown current drive mechanism by EC. Due to the lack of diagnostics data of those discharges, such as CES or MSE, we will focus on #26381, shown in Fig. 1, for detailed analysis.

**Fig. 4:**
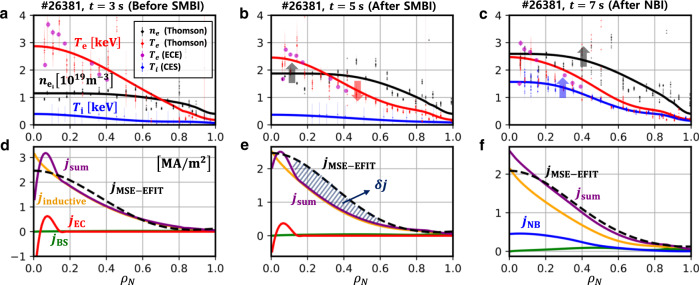
Kinetic profiles and current density profiles in #26381. Electron density (${{n}}_{{{{{{\rm{e}}}}}}}$) and temperature (${{T}}_{{{{{{\rm{e}}}}}}}$) and ion temperature (${{T}}_{{{{{{\rm{i}}}}}}}$) at ${t}{=}{3}\,{{{{{\rm{s}}}}}}$ (**a**), ${5}\,{{{{{\rm{s}}}}}}$ (**b**), and ${7}\,{{{{{\rm{s}}}}}}$ (**c**). Inductive (${{j}}_{{{{{{\rm{inductive}}}}}}}$), neutral beam injection-driven (${{j}}_{{{{{{\rm{NB}}}}}}}$), electron cyclotron-driven (${{j}}_{{{{{{\rm{EC}}}}}}}$), bootstrap (${{j}}_{{{{{{\rm{BS}}}}}}}$) current density, and summation of each component of the current density (${{j}}_{{{{{{\rm{sum}}}}}}}$) at ${t}{=}{3}\,{{{{{\rm{s}}}}}}$ (**d**), ${5}\,{\rm {{s}}}$ (**e**), and ${7}\,{{{{{\rm{s}}}}}}$ (**f**). Current density profiles by MSE-constrained EFIT (${{j}}_{{{{{{\rm{MSE}}}}}}{-}{{{{{\rm{EFIT}}}}}}}$) are also shown for comparison. The abscissa is the normalized toroidal magnetic flux, ${{\rho }}_{{{{{{\rm{N}}}}}}}$. The anomalous current (${\delta }{j}$) is indicated with the shade.

Figure 4a–c show 1-dimensional kinetic profiles fitted from the measured data at $t=3,\,5$ and $7\,{{{{{\rm{s}}}}}}$ in #26381 and Fig. 4d–f show the current density profiles obtained from the MSE-constrained EFIT and the interpretive analysis with TRIASSIC. Before SMBI was applied, the total current density profile is dominated by the inductive current and agrees with that from the MSE-EFIT. The difference at the center where EC exerts is mainly due to uncertainties of the MSE measurement near the axis. However, as the plasma density increased and the electron temperature decreased after SMBI, there appears a significant anomalous current density gap ($\delta j$ in Fig. 4e) between the MSE-EFIT and the simulation in the off-axis region. This anomalous off-axis current drive can be related to the ${l}_{{{{{{\rm{i}}}}}}}$ decrease shown in Fig. 1b. After NBI was applied instead of EC, the ion temperature and the plasma density increased, then the anomalous current gap almost disappears again. There are several uncertainties in the interpretive analysis such as assumptions of the effective ion charge, ${Z}_{{{{{{\rm{eff}}}}}}}$, neoclassical transport models, and fitting of kinetic profiles. We scanned the effective charge along $1.2\le {Z}_{{{{{{\rm{eff}}}}}}}\le 3.0$ with different radial profile shapes^21^, tried other neoclassical models^22,23^ and modified the kinetic profile fittings in several different ways, but the discrepancy has persisted nevertheless.

### The possibility of turbulence-driven current

Since the anomalous current density appears mainly at the off-axis region where the electron temperature gradient (ETG) is high, as shown in Fig. 4b, there is a possibility that the micro-instabilities such as the trapped electron mode (TEM) or the ETG mode could drive currents in this region. It is noteworthy that previous electrostatic gyrokinetic simulations have revealed that turbulence propagating in the electron-diamagnetic direction can generate electron momentum, which induces the toroidal current in the tokamak^2,4^.

**Fig. 5:**
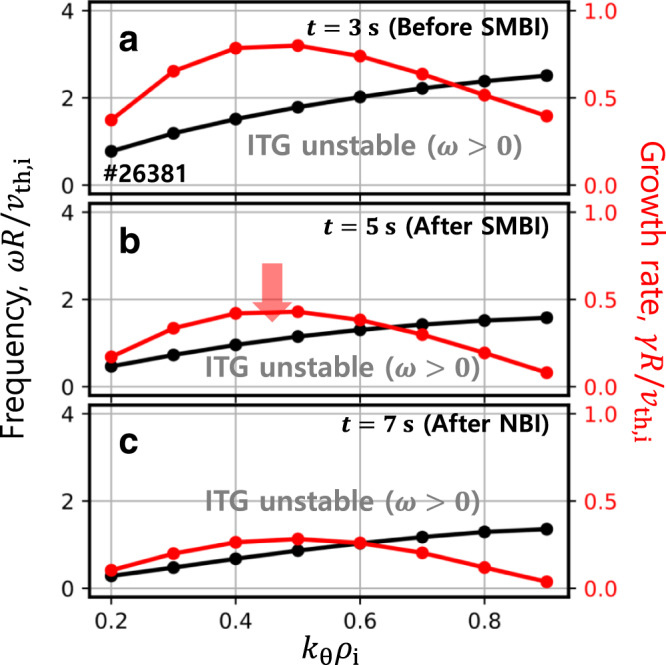
Linear gyro-kinetic analysis by GKW at *ρ*_N_ = 0.4 in #26381. Normalized frequency (${\omega }{R}{/}{{v}}_{{{{{{\rm{th}}}}}}{,}{\rm {{i}}}}$) and growth rate (${\gamma }{R}{/}{{v}}_{{{{{{\rm{th}}}}}}{,}{i}}$) at ${t}{=}{3}\,{{{{{\rm{s}}}}}}$ (**a**), ${5}\,{{{{{\rm{s}}}}}}$ (**b**), and ${7}\,{{{{{\rm{s}}}}}}$ (**c**). Here, ${\omega }$ is the mode frequency, ${R}$ is the major radius, ${{v}}_{{{{{{\rm{th}}}}}}{,}{{{{{\rm{i}}}}}}}$ is the ion’s thermal velocity, and ${\gamma }$ is the linear growth rate of the mode.

**Fig. 6:**
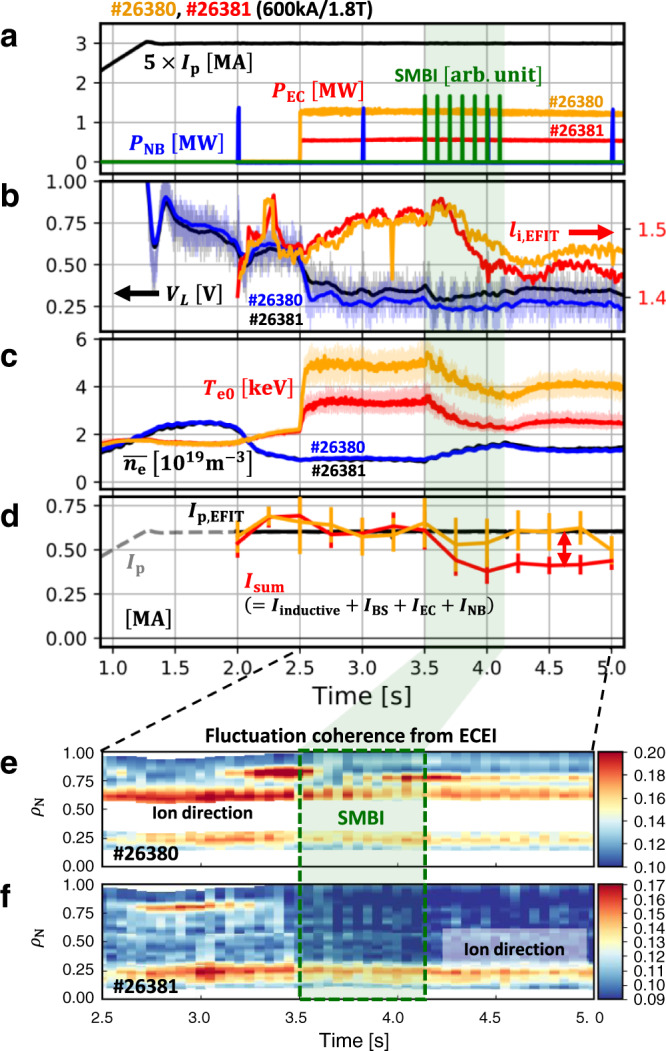
Comparison of main parameters between shot #26380 and #26381. **a** Plasma current (${{I}}_{{{{{{\rm{p}}}}}}}$), neutral beam injection (${{P}}_{{{{{{\rm{NB}}}}}}}$) and electron cyclotron (${{P}}_{{{{{{\rm{EC}}}}}}}$) power, and supersonic molecular beam injection (SMBI). **b** Loop voltage (${{V}}_{{{{{{\rm{L}}}}}}}$) and internal inductance (${{l}}_{{{{{{\rm{i}}}}}}}$). **c** Central electron temperature (${{T}}_{{{{{{\rm{e}}}}}}{0}}$) and line-averaged density ($\bar{{{n}}_{{{{{{\rm{e}}}}}}}}$). **d** Comparison of the total current obtained by interpretive simulations. Electron temperature fluctuation coherence was measured by the electron cyclotron emission imaging (ECEI) system for #26380 (**e**) and #26381 (**f**). The vertical shade in light green represents the time period where SMBI is applied.

Figure 5 shows the linear properties of the dominant micro-instability at ${\rho }_{{{{{{\rm{N}}}}}}}=0.4$, where the ETG is high, calculated by a gyrokinetic code, GKW^24^, for three phases in #26381. It shows that the electron turbulence is sub-dominant during the whole discharge, and instead, the ion temperature gradient (ITG) modes are the most unstable. This happens despite ${T}_{{{{{{\rm{e}}}}}}}$-profile peaking, probably due to the favorable role of high ${T}_{{{{{{\rm{e}}}}}}}/{T}_{{{{{{\rm{i}}}}}}}$ for the ITG excitation^25^. Those ITG modes become slightly mitigated after SMBI was applied. The fluctuation data measured by the electron cyclotron emission imaging (ECEI) system^26^ shown in Fig. 6f exhibits similar trends with the linear stability analysis. Note that the fluctuation coherence data in Fig. 6 are obtained by integrating the fluctuations measured by ECEI in the range of 0–250 kHz, which have a spatial resolution of 1–2 cm corresponding to the ITG and the TEM turbulence scale. Before SMBI was applied, fluctuations propagating in the ion-diamagnetic direction were observed prominently in the core, and subdominant in the edge region. After SMBI, the core fluctuation was mitigated and the edge fluctuation vanished, whereas the anomaly in the current appeared. This indicates that the anomalous current observed after SMBI is not likely to be driven by the long-wavelength turbulence measured by ECEI.

Another experimental evidence that the observed current could not be driven by turbulence detectable by the ECEI is presented in Fig. 6. The two shots, #26380 and #26381 are identical but for the EC heating power; 1.3 MW in #26380 and 0.6 MW in #26381. In #26380, due to the higher EC heating, the electron temperature is higher and the resulting turbulent fluctuations are also stronger, as shown in Fig. 6c, e, and f, respectively. However, the anomalous current gap between the interpretive simulation and the measurement is considerable in the lower EC heating case, #26381 (Fig. 6d). There is no new appearance of a fluctuation and the existing fluctuations become even diminished when the anomalous current drive occurs.

**Fig. 7:**
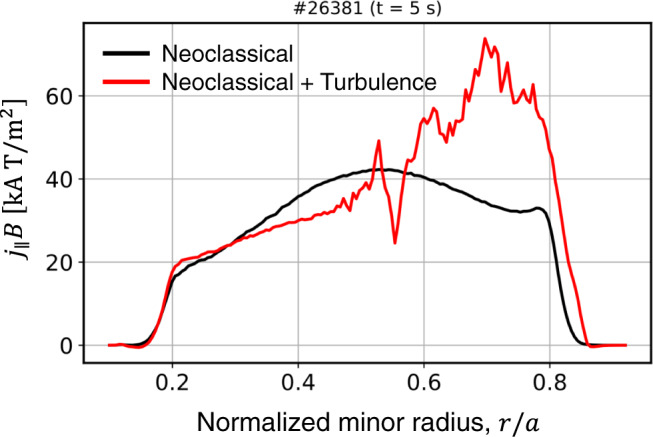
Current density profile estimated by the global nonlinear gyrokinetic simulation. The red and black curves represent the current density with and without considering the trapped electron mode (TEM), respectively.

As mentioned before, the anomalous current observed in this experiment is about 30% of the total plasma current. Previous studies report that the ETG-driven current is distributed with a corrugated profile with a net radial integration value almost vanishing^2^, and the TEM-driven current is reported to be somewhat lower than the bootstrap current level^4^. We have calculated the amount of the TEM-driven current in #26381 using a nonlinear gyrokinetic solver, GTS^4,27^. Figure 7 shows the self-generated current at $t=5.0\,{{{{{\rm{s}}}}}}$ in #26381, with (red) and without (black) considering the turbulence effect. The level of the additional current driven by the turbulence is larger than that in the previous study^4^ and driven in the off-axis region. However, considering that the bootstrap current in #26381 is <5% of the total current, the additional current by the turbulence is not high enough to explain the amount of anomalous current observed in our experiment, about 30% of the total current. The observed anomalous current is comparable to the NB-driven current. We also checked the possibility of another current source, pressure-driven currents^28,29^, to understand the current but those are also found to be negligible in the KSTAR geometry.

We do not yet clearly understand the mechanism of this phenomenon, and therefore, we only introduced the dedicated experiments in moderate-performance plasma regimes in which other known dominant current sources can be excluded for identification of the observed current source. A clarification of the physical mechanism of this anomalous current drive is necessary for our future exploration of high-performance plasma regimes in which this phenomenon can possibly help develop non-inductive steady-state operation scenarios in a tokamak.

In this communication, we have reported the clear observation of a new current drive source in a tokamak. In dedicated experiments with minimized known external and self-generated current drives, we observed an anomalous current gap that cannot be explained by the present theories. This is a robust phenomenon that is observed in plasmas with fuelling. Our studies have not found any supporting evidence for the existing current drive mechanisms so far including those related to micro-instabilities. For the identification of the mechanism, one needs to extend turbulence measurements to a broader ${k}_{\perp }$-range including shorter wavelengths and perform further global nonlinear gyrokinetic simulations followed by detailed analyses. Zonal flows which regulate turbulence and nonlocal phenomena^30^ may play a role. In addition, a nonuniform ${Z}_{{{{{{\rm{eff}}}}}}}$-profile may contribute to a current anomaly by changing the turbulent characteristics, although we did not observe its significant effect on the neoclassical properties. It may also be related to the ion rotation reversal depending on turbulent characteristics^19^ though its correlation with the anomalous current is not clear yet. A possible contribution from runaway electrons needs to be addressed as well. In order to actively apply this current drive mechanism for the steady-state operation of a tokamak, it is required to develop a theory that can explain this phenomenon and validate it against experiments.

## Methods

### KSTAR

The Korean superconducting tokamak advanced research (KSTAR)^10^ is a magnetic confinement fusion facility based on the tokamak concept operated by the Korea Institute of Fusion Energy (KFE) in Daejeon, Republic of Korea. The magnetic system of KSTAR consists of 16 toroidal field coils and 14 poloidal field coils made of superconducting magnets. The toroidal magnetic field strength is up to 3.5 T and the plasma current is up to 1 MA. The major and minor radii are 1.8 and 0.5 m, respectively.

### Experimental setup

Our experiments in KSTAR were carried out with two main auxiliary heating systems, the electron cyclotron (EC) heating and the neutral beam injection (NBI). EC heating is the main tool to heat the electrons in a tokamak by using EC resonance waves. The second harmonic X-mode EC heating system is equipped with a gyrotron that generates up to the power of 0.8 MW at the frequency of 105 GHz in KSTAR. The NBI system in KSTAR injects the Deuterium beam of the energy of $\lesssim 100\,{{{{{\rm{keV}}}}}}$ into the plasma and produces fast ions in the plasma. The fast ions injected in the toroidal direction heat the plasma and also generate the electric current. Deuterium gas fueling can be done by supersonic molecular beam injection (SMBI) and piezoelectric valve at D-port (PVD). SMBI is a gas injection system arranged for perpendicular injection with the purpose of particle fueling and instability mitigation, and PVD is an effective fueling system that supplies the Deuterium gas from the diverter region in KSTAR.

### Numerical setup

The interpretive simulation is conducted with an integrated suite of codes, TRIASSIC^16^ by using the kinetic profiles measured in the experiments. In TRIASSIC, the 1.5-dimensional transport analysis is performed with ASTRA^31^ and the plasma resistivity ($\eta$) and the bootstrap current are obtained by NCLASS^32^. The heating and current drive by NBI and EC are calculated by NUBEAM^33^ and TORAY^34^, respectively. The plasma equilibria are reconstructed by EFIT^17^ with a constraint of motional Stark effect (MSE) measurement^18^. The micro-stability is analyzed with a gyrokinetic code, GKW^24^ and GTS^27^. During the main numerical simulations, the effective charge is assumed ${Z}_{{{{{{\rm{eff}}}}}}}=2.0$ with the Carbon impurity only. Additional simulations to verify the effect of ${Z}_{{{{{{\rm{eff}}}}}}}$ were also done with different radial profile shapes^21^ within $1.2\le {Z}_{{{{{{\rm{eff}}}}}}}\le 3.0$.

### Code descriptions

EFIT^17^: EFIT is an efficient numerical method for self-consistent reconstruction of the internal current profiles and their associated magnetic topology using the constraints from external diagnostic measurements.

TRIASSIC^16^: TRIASSIC is an integrated suite of codes for simulation and computation with various purposes of tokamak plasma analyses. It contains multiple plasma simulation codes, including equilibrium solvers, 1D/2D plasma transport solvers, and neoclassical/anomalous transport, plasma heating/cooling, and cold neutral models.

ASTRA^31^: An automated system for transport analysis, ASTRA, numerically solves a set of equations describing the transport of the particles, momentum, thermal energy, and the magnetic flux in the flux-coordinate in a tokamak.

NCLASS^32^: NCLASS gives a comprehensive treatment of neoclassical effects in an arbitrary collisionality and geometry of a tokamak. It deals with a set of fluid force balance equations for multiple species to evaluate plasma conductivity, bootstrap current, and other features of neoclassical transport.

NUBEAM^33^: The NUBEAM module is a comprehensive computational model for NBI and fusion reactions in an arbitrary geometry of tokamaks. It uses the Monte-Carlo method to compute the power deposition, driven current, momentum transfer, fueling, and fast ion profiles by NBI in tokamak plasmas.

TORAY^34^: TORAY is a ray-tracing code for analyzing the EC heating and current drive in the toroidal geometry.

GKW^24^: A gyrokinetic code, GKW, is used to study the turbulent transport and stability problems arising in tokamak plasmas. It solves the gyrokinetic equation on a fixed grid in the 5D space using a combination of the finite difference and the pseudo-spectral method.

GTS^27^: GTS is a global nonlinear gyrokinetic solver based on a generalized gyrokinetic model that self-consistently couples neoclassical and turbulent dynamics. It provides the perturbed quantities by the electron-scale turbulence, such as the radial flux and the current density.

## Data Availability

Raw data were generated from the KSTAR team. The data supporting the findings of this work are available from the corresponding author upon request.
